# Leptin in the canine uterus and placenta: possible implications in pregnancy

**DOI:** 10.1186/s12958-015-0003-6

**Published:** 2015-03-08

**Authors:** Orsolya Balogh, Livia P Staub, Aykut Gram, Alois Boos, Mariusz P Kowalewski, Iris M Reichler

**Affiliations:** Clinic of Reproductive Medicine, Vetsuisse-Faculty, University of Zurich, Winterthurerstrasse 260, 8057 Zurich, Switzerland; Institute of Veterinary Anatomy, Vetsuisse-Faculty, University of Zurich, Winterthurerstrasse 260, 8057 Zurich, Switzerland

**Keywords:** Leptin, Leptin receptor, Dog, Gestation, Uterus, Placenta, Immunohistochemistry, *In situ* hybridization

## Abstract

**Background:**

Leptin (Lep) is known for its involvement in the regulation of reproductive functions. It is important for uterine receptivity, implantation, placental growth and maternal energy homeostasis in several species, but Lep’s function in the pregnant dog has not been investigated.

**Methods:**

Pregnant bitches were ovariohysterectomized at pre-implantation, post-implantation, mid-gestation and prepartum luteolysis. Two additional groups were treated with aglepristone in mid-gestation, and ovariohysterectomized 24 or 72 h later. Lep and leptin receptor (LepR) gene expression was detected by semi-quantitative real-time PCR in pre-implantation and inter-placental uterine sections (Ut) and in utero-placental compartments (Ut/Pl). Immunohistochemistry and in situ hybridization (ISH) were performed for Lep and LepR protein and mRNA localization. Parametric one-way ANOVA, paired *t*-test and Wilcoxon signed-rank test were used for statistical analysis.

**Results:**

In the Ut/Pl, Lep expression was higher at post-implantation and prepartum luteolysis than at mid-gestation, while in the Ut, Lep mRNA levels did not change during pregnancy. LepR expression in the Ut/Pl was up-regulated at prepartum luteolysis compared to the earlier stages. In the Ut, highest LepR mRNA was found at pre- and post-implantation. LepR expression was down-regulated in the Ut/Pl compared to the Ut at post-implantation and at mid-gestation. Aglepristone treatment resulted in a decrease of Lep mRNA levels from 24 to 72 h in the Ut without concomitant changes in the Ut/Pl or in LepR levels. Lep and LepR immunoreactivities were strong in the luminal and glandular epithelium in the Ut with abundant LepR signals in the subepithelial stroma. In the Ut/Pl, fetal trophoblasts stained stronger for Lep and LepR than decidual cells, and signals for both proteins were also detected in the glandular chambers. The myometrium, blood vessel media, and sporadically also the endothelium stained for Lep and LepR. ISH showed similar signal distribution in the Ut and Ut/Pl.

**Conclusions:**

Lep and LepR are differentially expressed in the canine uterus and placenta during pregnancy, and their presence in various cell types indicates paracrine/autocrine roles. The Lep signaling system may be one of the pathways involved in feto-maternal cross-talk, implantation and maintenance of pregnancy, and may have a regulatory role around parturition.

## Background

Leptin (Lep) was originally thought to be secreted exclusively by adipose tissue, regulating energy metabolism and satiety [1]. Soon thereafter it became clear that Lep affects reproductive processes both centrally and peripherally, and its local expression within reproductive tissues also supports paracrine/autocrine actions through its receptors [2-7]. During gestation, Lep concentrations in maternal serum are significantly increased in, *e.g.*, women, rats, mice and mares [8-12] and decrease after birth, indicating a physiological role in pregnancy. Besides adipose tissue, human, rat and murine placentae also express Lep [10,13,14], but it seems that the placental contribution to circulating levels is only functionally significant in women [7]. Lep regulates various processes that are crucial for placental development and function including nutrient transfer across and within the placenta [15,16]. Additionally, Lep has also angiogenic properties [17,18].

Fetal trophoblast cells invade the maternal endometrium in species with invasive placentation like humans, rodents, and to some extent also in dogs [19]. Lep promotes trophoblast invasion in humans by acting on matrix metalloproteinase 2 (MMP-2) and MMP-9, which are regarded as key enzymes for successful implantation [20,21]. *In vitro*, Lep stimulated MMP-2 secretion and the activity of MMP-2 and MMP-9 from human cytotrophoblasts [22,23] as well as invasion by day 10 murine trophoblast cells [24]. Furthermore, already in early pregnancy, both at the pre-implantation phase and during the window of implantation, Lep was shown to regulate uterine receptivity and embryo development in species other than the dog [25-30]. Human endometrial epithelial cells treated with Lep *in vitro* expressed higher levels of β3 integrin, leukemia inhibitory factor (LIF), interleukin-1 (IL-1) and their receptors [25,26], which facilitate embryo adhesion and have immuno-modulatory roles during embryo-maternal interaction. It seems that the developing pre-implantation embryo is under the local influence of maternal Lep in the uterus and can respond to it, as LepR expression was found in oocytes and embryos from the 2-cell to 8-cell or blastocyst stages [27-29]. Lep increased cell numbers and blastocyst formation rates in murine and ovine embryos [27,28], but reduced quality and developmental rate in horses [29].

In dogs, maintenance of pregnancy is dependent on an adequate supply of progesterone (P4), the sole source of which is the corpus luteum (CL), as the placenta is devoid of steroidogenic activity [31-33]. The luteal phase in pregnant and non-pregnant dogs is comparable in length and in peripheral levels of P4, estrogens and prolactin (PRL), and only relaxin concentrations are different since it is produced by the placenta [32,34-36]. Therefore, other endocrine and molecular mechanisms may already be crucial early on to support survival of the embryo and to maintain a successful pregnancy. During early canine embryo-maternal contact, differential gene expression of members of the prostaglandin synthesis pathway, growth factors, cytokines, immune cell receptors, steroid hormone receptors and the PRL receptor points to the role of the blastocyst to interact with the uterine milieu to signal its presence [37-40]. Some of the above-mentioned factors are also expressed at the time of implantation-placentation, as shown by Beceriklisoy et al. [39], Kowalewski et al. [41,42] and Gram et al. [43].

Lep’s role in canine reproduction and especially during pregnancy is not extensively studied and is poorly understood, although the positive relationship with short- and long-term energy status and obesity is well known [44-46]. Lep is produced in proportion to the amount of fat depots, and its peripheral concentration correlates with adiposity regardless of gender, breed, age and neuter status [46]. However, Saleri et al. [47] measured higher levels of Lep in female than in male dogs, which also varied according to the stage of the reproductive cycle. Up-regulation of Lep gene expression in the CL during the early luteal phase in non-pregnant bitches [48], and the presence of Lep immunoreactive protein in luteal cells and in granulosa cells of mature luteinized follicles [49], may indicate a positive action of Lep on follicular and luteal function and perhaps on steroid hormone production in the dog. To date, Lep expression in the canine uterus was only examined in the first half of gestation using qualitative PCR, which yielded negative results [37,39]. Bartel et al. [50] found Lep and LepR immunoreactivity in the uteri of non-pregnant bitches, but pregnant dogs were not studied.

The role of Lep in canine uterine and placental function during gestation is still not clear and has not been investigated in detail. We hypothesize that the canine placenta is a source of Lep, and that Lep signaling may be involved in the regulation of pregnancy establishment and maintenance. Therefore, in this study, we determined relative gene expression of Lep and LepR in full-thickness uterine (Ut) and utero-placental (Ut/Pl) sections of bitches from various stages of pregnancy and after aglepristone-induced abortion in mid-gestation; the latter experiment was to compare to changes occurring during parturition. Additionally, Lep and LepR protein and mRNA were localized by immunohistochemistry (IHC) and *in situ* hybridization (ISH), respectively, in representative sections of the Ut and Ut/Pl during gestation.

## Methods

All uterine and utero/placental tissues used in this study were collected in the framework of previous studies [41,51], in accordance with the appropriate animal welfare legislations. Animal experiments were approved by the respective ethics committees and conducted under permit no. II 25.3-19c20-15c GI 18/14 and VIG3-19c20/15c GI 18,14 (Justus-Liebig University, Giessen), and permit no. Ankara 2006/06 (Faculty of Veterinary Medicine, University of Ankara).

### Animals

Healthy bitches (2–8 years, different breeds) were mated 2 days after ovulation (serum P4 ≥ 5 ng/mL) [52]. Day of mating was designated as day 0. Dogs were ovariohysterectomized on d 8–12 (pre-implantation, n = 5), d 18–25 (post-implantation; n = 5), d 35–40 (mid-gestation; n = 5) and during prepartum luteolysis (n = 3; defined as P4 < 3 ng/ml in two consecutive serum samples taken 6 h apart starting from d 58). Additionally, bitches on d 40–45 of gestation were treated with the progesterone-receptor blocker aglepristone (Alizin®, Virbac, Carros Cedex, France; 10 mg/kg BW subcutaneously twice, 24 h apart) to induce abortion, and were ovariohysterectomized 24 or 72 h after the last injection (n = 4 per group). At the pre-implantation stage, pregnancy was confirmed by detecting embryos in uterine flushes immediately after surgery.

### Tissue samples

Full thickness Ut samples were collected from bitches in the pre-implantation group. From the post-implantation stage until prepartum luteolysis, full thickness Ut/Pl and inter-placental Ut sections were collected. Inter-placental Ut was not available from the prepartum luteolysis group. For preservation of RNA, all tissues were incubated overnight in RNAlater® at 4°C (Ambion Biotechnologie GmbH, Wiesbaden) and afterwards stored at −80°C. For IHC and ISH, all tissues were fixed in 10% neutral phosphate-buffered formalin at 4°C for 24 h, washed daily in phosphate buffered saline for one week, dehydrated in a graded ethanol series and embedded in paraffin-equivalent Histo-Comp (Vogel, Giessen, Germany) or paraffin [41,53].

### Semi-quantitative real-time (TaqMan) PCR

After isolation of total RNA using TRIzol® Reagent (Invitrogen, Carlsbad, CA, USA), 100–200 ng of RNA per sample was DNAse treated (RQ1 RNase-free DNase; Promega, Dübendorf, Switzerland) to eliminate genomic DNA contamination according to the manufacturer’s instructions and as previously described [54]. Reverse transcription was carried out in an Eppendorf Mastercycler Thermal Cycler (Vaudaux-Eppendorf AG, Basel, Switzerland) using reagents from Applied Biosystems (Foster City, CA, USA) according to our protocols [54]. Semi-quantitative real-time (TaqMan) PCR for the detection of canine Lep [GenBank: NM_001003070] and LepR [GenBank: NM_001024634; only the long isoform is known] was carried out using the same primers and TaqMan probes as previously reported by our group [48], *i.e.*,: Lep (forward): 5’-GGG TCG CTG GTC TGG ACT T-3’, Lep (reverse): 5’-CTG TTG GTA GAT GGC CAA CGT-3’, Lep TaqMan probe: 5’-TCC TGG GCT CCA ACC AGT CCT GAG T-3’; LepR (forward): 5’-CAT TTG CGG AGG GAT GGT T-3’, LepR (reverse): 5’-AGC GGT TTC ACC ACG GAA T-3’, LepR TaqMan probe: 5’-TTG ACT CTT CAC CAA CGT GTG TGG TTC C-3’. TaqMan probes were labeled at the 5’-end with the reporter dye 6-carboxyfluorescein (FAM), and at the 3’-end with the quencher 6-carboxytetramethyl-rhodamine (TAMRA). Reactions were carried out in an automated fluorometer (ABI PRISM_TM_ 7500 Sequence Detection System, Applied Biosystems, Darmstadt, Germany) using 96-well optical plates following our protocols [55,56]. Samples were run in duplicates. Canine GAPDH [GenBank: AB028142] (GAPDH (forward): 5’-GCT GCC AAA TAT GAC GAC ATC A-3’, GAPDH (reverse): 5’-GTA GCC CAG GAT GCC TTT GAG-3’, GAPDH TaqMan probe: 5’-TCC CTC CGA TGC CTG CTT CAC TAC CTT-3’) [54] and cyclophyllin A (Prod. No. Cf03986523-gH, Applied Biosystems, Foster City, CA, USA) served as reference genes, and autoclaved water instead of cDNA was used as negative control.

### Immunohistochemistry

An indirect immunoperoxidase method was used on tissue samples collected from pregnant dogs according to Kowalewski et al. [54]. For the detection of Lep, a rabbit polyclonal affinity purified antibody directed against the N-terminal region of the human leptin (ARP41697_P050, Aviva Systems Biology, San Diego, USA) diluted 1:200 was used, and for LepR, a goat polyclonal affinity purified antibody raised against a peptide mapping at the C-terminus of the short form of LepR of mouse origin and recommended for the detection of both short and long LepR isoforms (Ob-R (M-18): sc-1834, Santa Cruz Biotechnology Inc., CA, USA) diluted 1:50. Briefly, 2–3 μm thick sections of Ut/Pl and uterine tissues were mounted on SuperFrost Plus microscope slides (Menzel-Gläser, Braunschweig, Germany), deparaffinized in xylol and rehydrated in a graded ethanol series. Antigen retrieval was achieved by heat induction, incubating slides in 10 mM citrate buffer (pH 6.0) for 15 min at 100°C. Endogenous peroxidase activity was quenched with 0.3% hydrogen peroxide in methanol. Non-specific binding sites were blocked with 10% goat serum (for Lep; KPL, Gaithersburg, USA) or 10% horse serum (for LepR; Vector Laboratories, Burlingame, USA). Slides were incubated with the primary antibodies overnight at 4°C. Biotinylated goat anti-rabbit IgG and horse anti-goat IgG (Vector Laboratories, Burlingame, USA) at a dilution of 1:100 were used as secondary antibodies for Lep and LepR, respectively. Signals were enhanced using the avidin/biotinylated peroxidase complex (Vectastain ABC Kit, Vector Laboratories, Burlingame, USA) and color reactions were achieved by applying 3,3’-diaminobenzidine (DAB) as chromogen substrate (Liquid DAB+ substrate Kit, Dako Schweiz AG, Baar, Switzerland). Finally, slides were counterstained with Mayer’s hematoxylin, dehydrated in a graded ethanol series and covered with coverslips. Isotype controls were carried out with pre-immune rabbit (for Lep) and goat (for LepR) IgG (Vector Laboratories, Burlingame, USA). Murine ovaries with corpora lutea were used as positive controls for both Lep and LepR.

### *In situ* hybridization (ISH)

A non-radioactive method [55] was used for tissue localization of Lep and LepR mRNA in representative samples of Ut and Ut/Pl. Templates for cRNA probe synthesis were generated by the following canine-specific primers: Lep (forward) - 5’-ATG CGT TGT GGA CCT CTG TG-3’, Lep (reverse) - 5’-GGT TGG AGC CCA GGA ATG AA-3’, amplicon length 203 bp; LepR (forward) - 5’-CAT GGT GGG TGA CCG TGT TA-3’, LepR (reverse) - 5’-TCC CTC GAG TGA TTG GAT TGC-3’, amplicon length 232 bp. PCR products were separated on a 2% ethidium bromide-stained agarose gel, purified with the Qiaex II gel extraction system (Qiagen GmbH, Hilden, Germany) and cloned into a pGEM-T plasmid (Promega, Dübendorf, Switzerland). The plasmid clones containing the inserts were digested with restriction enzymes *Nco*I and *Not*I (New England Biolabs, Frankfurt, Germany) to obtain antisense and sense cRNA probes, respectively, which were labelled with digoxigenin (DIG-RNA labelling kit, Roche Diagnostics AG, Rotkreuz, Switzerland). Dot-blot analysis was carried out with serially diluted DIG-labeled cRNA on a positively charged nylon membrane (Roche Diagnostics) for semi-quantitation of labeled cRNA concentration. Paraffin-embedded cross sections of Ut and Ut/Pl were dewaxed, rehydrated, digested with 70 μg/ml proteinase K (Sigma-Aldrich Chemie GmbH) and post-fixed with 4% paraformaldehyde. Hybridization was carried out overnight at 37°C in a formamide chamber. Signals for Lep and LepR mRNA were detected by DIG-labelled cRNA probes and alkaline phosphate-conjugated sheep anti-DIG Fab fragments (Roche Diagnostics AG, Rotkreuz, Switzerland) diluted 1:5000 in 1% ovine serum. Color visualization was performed with 5-bromo-4-chloro-3-indolyl phosphate in the presence of nitroblue tetrazolium (NBT/BCIP; Roche Diagnostics AG, Rotkreuz, Switzerland).

### Statistical analysis

Only valid data on Lep and LepR gene expression were considered, where the relative amount of reference genes for a sample were constant (*i.e.*, similar in the duplicate samples). Relative gene expression (RGE) was calculated by the ΔΔCt method as described [54,56]. Logarithmic transformation was performed when observed data were not normally distributed (Kolmogorov-Smirnov test, P < 0.05). A parametric one-way ANOVA followed by Tukey Honestly Significant Difference was used to compare RGE of Lep and LepR among pregnancy stages and after aglepristone treatment. We performed paired *t*-test or Wilcoxon signed-rank test for the comparison of Lep or LepR mRNA expression between the Ut/Pl and the Ut in the post-implantation and mid-gestation stages. Results are presented as mean ± standard deviation of observed data or geometric mean ± deviation factor in the case of ln-transformed data. Level of significance was set at P ≤ 0.05; statistical calculations were carried out with IBM® SPSS® Statistics for Windows, Version 19.0 (Armonk, NY, USA).

## Results

### Lep in the Ut/Pl and Ut

#### Lep gene expression during pregnancy and after aglepristone induced abortion

**Figure 1:**
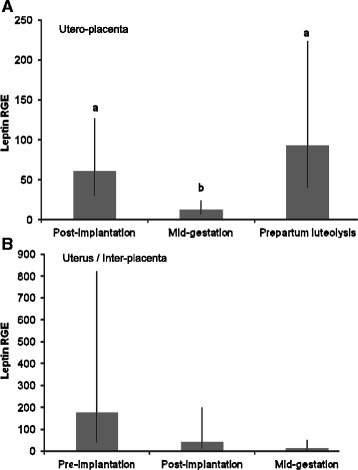
**Leptin gene expression in the utero-placental and uterine compartments during pregnancy.** Leptin gene expression during pregnancy in the utero-placental compartments **(A)**, and in the pre-implantation uterus and inter-placental uterine sections **(B)**. RGE: relative gene expression. Bars and whiskers present the geometric mean and the deviation factor. Bars with different superscripts differ at P ≤ 0.043.

**Figure 2:**
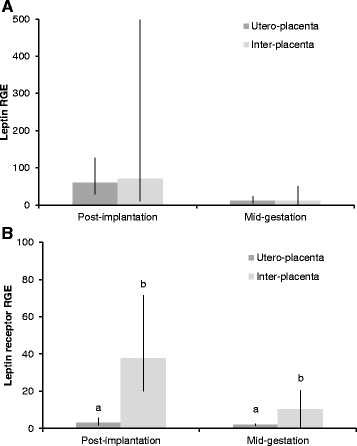
**Comparison of leptin and leptin receptor gene expression between utero-placental and inter-placental uterine sections.** Relative gene expression of leptin **(A)** and leptin receptor **(B)** between utero-placental and inter-placental uterine sections at post-implantation and at mid-gestation. RGE: relative gene expression. Bars and whiskers present the geometric mean and the deviation factor, or the mean and standard deviation of observed data in case of mid-gestation leptin receptor gene expression. Bars with different superscripts within the same pregnancy stage differ at P = 0.003 (post-implantation) and P = 0.043 (mid-gestation).

In the Ut/Pl, we found lower Lep mRNA concentrations at mid-gestation than at post-implantation and prepartum luteolysis (P ≤ 0.043; Figure 1A). In the Ut, Lep expression did not change throughout gestation (P = 0.12; Figure 1B). Lep mRNA levels were similar between the Ut/Pl and Ut both at the post-implantation (P = 0.87) and mid-gestation stages (P = 1.00; Figure 2A).

**Figure 3:**
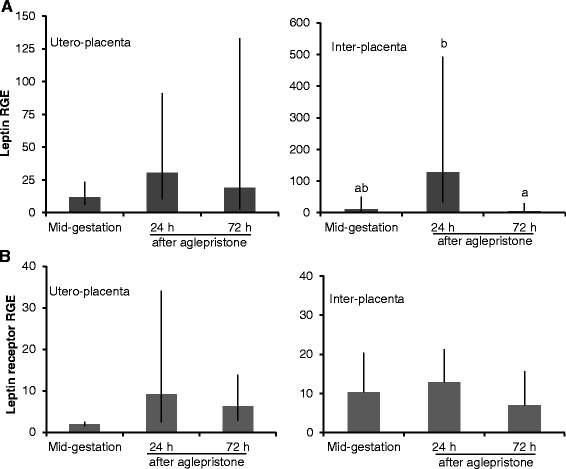
**Leptin and leptin receptor gene expression in the utero-placental and uterine compartments after abortion induced with aglepristone.** Leptin gene expression **(A)** in utero-placental compartments and inter-placental uterine sections at mid-gestation, 24 and 72 h after aglepristone administration. Leptin receptor gene expression **(B)** in the utero-placental compartments and inter-placental uterine sections at mid-gestation, 24 and 72 h after aglepristone administration. RGE: relative gene expression. Bars and whiskers present the geometric mean and the deviation factor or the mean and standard deviation of observed data in case of inter-placental leptin receptor gene expression. Bars with different superscripts differ at P = 0.05.

Induction of abortion with aglepristone in mid-gestation did not affect Lep gene expression in the Ut/Pl (P = 0.54), but lower levels were detected in the Ut at 72 h than at 24 h after the second aglepristone injection (P = 0.05; Figure 3A).

### Immunohistochemical detection of Lep during pregnancy

**Figure 4:**
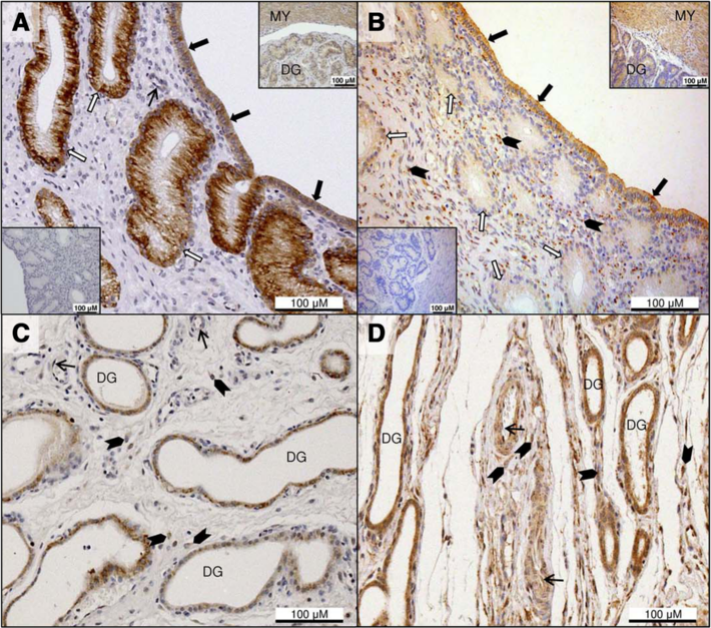
**Immunohistochemical detection of leptin and leptin receptor in the pre-implantation uterus and inter-placental sections during pregnancy.** Immunohistochemical localization of leptin (Lep; **A**, **C**) and leptin receptor (LepR; **B**, **D**) in pre-implantation and inter-placental uterine sections (Ut) during pregnancy. **(A)** Lep immunostaining at pre-implantation is present in the luminal epithelium (solid arrows) and in the superficial glands (open arrows); endothelial cells stain occasionally (thin arrow). The myometrium and deep uterine glands also show positive reaction for Lep (inset upper right); inset (lower left) indicates the isotype control for Lep. **(B)** LepR immunoreactivity in the Ut at pre-implantation is noted in the surface epithelium (solid arrows), and in the endometrial stroma close to the lumen (solid arrowheads). Superficial glands (open arrows) stain weakly, and the myometrium and deep uterine glands also show positive signals (inset upper right); inset (lower left) shows the isotype control for LepR. **(C)** Strong Lep staining is visible in the deep uterine glands at mid-gestation; stromal signals are also evident (solid arrowheads), while endothelial cells show sporadic, weak signals (thin arrows). **(D)** At mid-gestation, the epithelium of deep uterine glands has stronger LepR immunoreactivity than at pre-implantation. Stromal signals are still present (solid arrowheads), and signals are also found occasionally in the endothelium (thin arrows). DG: deep uterine glands, MY: myometrium.

In the Ut, Lep immunoreactivity was detected in the surface epithelium and superficial endometrial glands (Figure 4A). Weaker positive signals were present in the epithelium of deep uterine glands at pre- and post-implantation, but at mid-gestation, they became stronger in the deep glands but were variably present in the endometrial stroma (Figure 4C).

**Figure 5:**
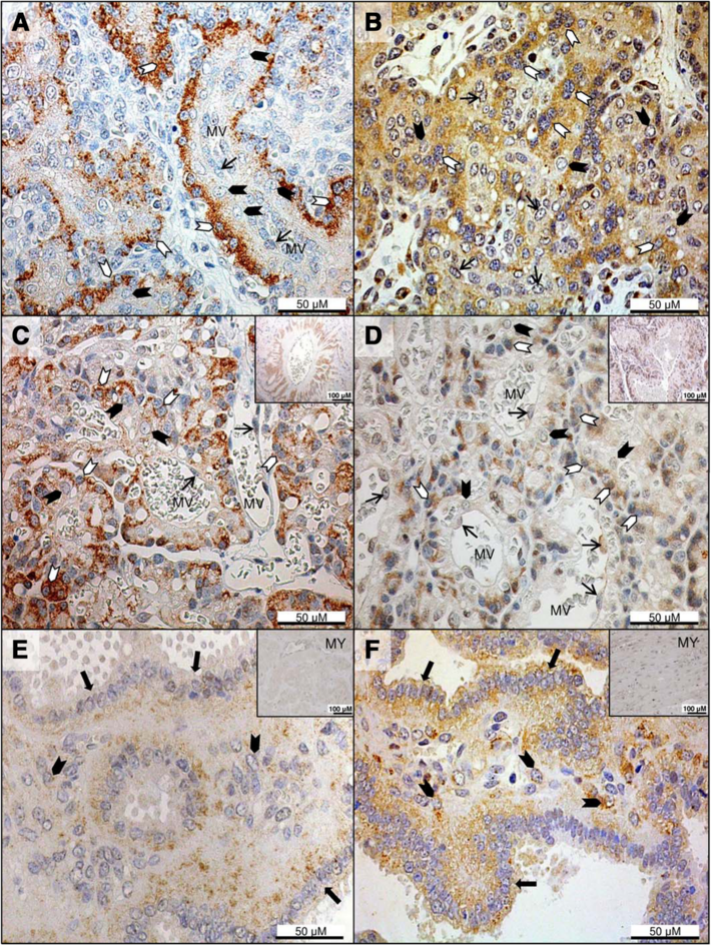
**Immunohistochemical detection of leptin and leptin receptor in the utero-placental compartments during pregnancy.** Immunohistochemical localization of leptin (Lep; **A**, **C**, **E**) and leptin receptor (LepR; **B**, **D**, **F**) in the utero-placental compartments (Ut/Pl) during pregnancy. **(A)** Lep signals are strong in fetal trophoblast cells (open arrowheads) of the placental labyrinth at mid-gestation, while maternal decidual cells (solid arrowheads) stain more weakly and blood vessel endothelial cells (thin arrows) show sporadic signals. **(B)** LepR positive trophoblasts (open arrowheads) are present in the placental labyrinth at mid-gestation. LepR immunoreactivity is less intense in decidual cells (solid arrowheads) and in blood vessel endothelium (thin arrows). **(C)** Strong Lep immunoreactivity is evident in fetal trophoblast cells (open arrowheads) of the placental labyrinth at prepartum luteolysis with weaker signals in decidual cells (solid arrowheads) and occasional staining in the endothelium (thin arrows). Intense signals are detected in trophoblasts at the base of the labyrinth invading large maternal vessels (inset). **(D)** LepR positive trophoblast cells (open arrowheads) are shown in the placental labyrinth at prepartum luteolysis with less intense staining in maternal decidual cells (solid arrowheads) and blood vessel endothelium (thin arrows). The inset shows positive reaction in fetal trophoblast cells invading large maternal vessels at the base of the labyrinth. **(E)** Lep staining is present in the epithelial cells of the glandular chambers (solid arrows) and sporadic signals are also visible in the stroma (solid arrowheads). Positive staining in the myometrium is shown in the inset. **(F)** In the glandular chambers, epithelial cells stain positive for LepR (solid arrows) and stromal signals (solid arrowheads) are also present. Positive immunoreactivity in the myometrium is presented in the inset. MV: maternal vessel, MY: myometrium.

In the Ut/Pl, fetal trophoblasts of the placental labyrinth stained positively for Lep, and maternal decidual cells had weaker signals at all pregnancy stages (Figure 5A,C). Invading trophoblast cells surrounding large maternal vessels at the base of the placental labyrinth showed intense immunoreactivity (Figure 5C inset). Epithelial cells of the glandular chambers stained weakly with signals present also in the stroma (Figure 5E).

Lep was also detected in smooth muscle cells of the myometrium (Figures 4A upper right inset and 5E inset) and of blood vessels, and sporadically in the endothelium (Figures 4A,C and 5A,C).

### In situ hybridization for Lep

**Figure 6:**
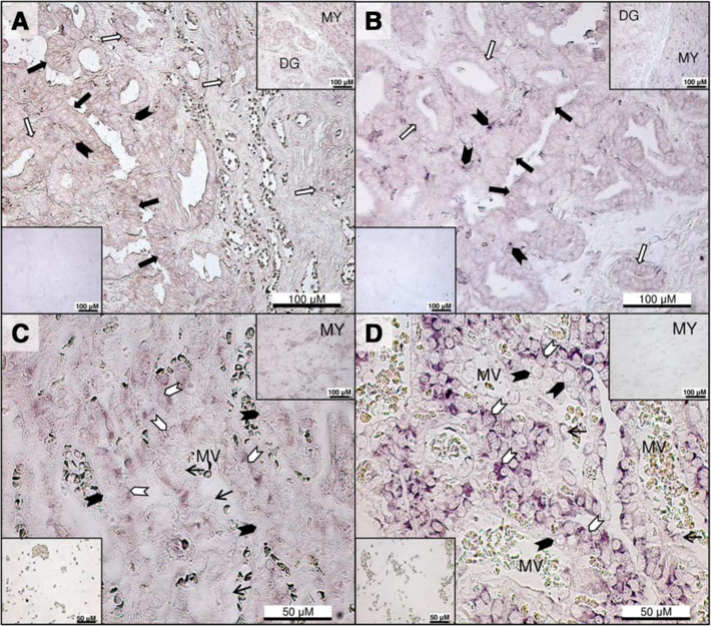
***In situ*** **hybridization for leptin and leptin receptor in the inter-placental uterus and utero-placental compartments during pregnancy.** Localization of leptin (Lep) and leptin receptor (LepR) mRNA by *in situ* hybridization in inter-placental sections (Ut) at post-implantation **(A, B)**, and in the utero-placental compartments (Ut/Pl) at prepartum luteolysis **(C, D)**. **(A)** Lep mRNA expression is evident in the surface epithelium (solid arrows) and superficial glandular epithelium (open arrows) with weak sub-epithelial stromal signals (solid arrowheads). Deep uterine glands and the myometrium also stain positively for Lep (inset upper right). A negative control is shown in the lower left inset. **(B)** LepR mRNA is expressed in the luminal epithelium (solid arrows) and superficial glandular epithelial cells (open arrows). Note the prominent stromal signals (solid arrowheads) close to the lumen. The upper right inset is positive staining for LepR in the deep uterine glands and myometrium, and the lower left inset shows a negative control. **(C)** Intense Lep expression is noted in the fetal trophoblast cells (open arrowheads) of the placental labyrinth at prepartum luteolysis, in contrast to weak staining in maternal decidual cells (solid arrowheads) and sporadic signals in blood vessel endothelium (thin arrows); the upper right inset shows positive reaction in the myometrium, and the lower left inset is the negative control sense probe for Lep. **(D)** LepR mRNA expression is strong in the placental labyrinth at prepartum luteolysis within fetal trophoblast cells (open arrowheads), while decidual cells have less intense signals (solid arrowheads) and endothelial cells (thin arrows) stain occasionally; the upper right inset shows LepR mRNA in the myometrium, and the lower left inset presents the negative control sense probe for LepR. DG: Deep uterine glands, MY: Myometrium, MV: maternal vessel.

In the Ut, Lep mRNA was found in the luminal and glandular epithelial cells of the endometrium (Figure 6A). In the Ut/Pl, fetal trophoblasts within the placental labyrinth gave stronger signals than decidual cells (Figure 6C), while the epithelium of the glandular chambers and deep uterine glands stained weakly (not shown). Strong positive signals were also detected in the myometrium (Figure 6A upper right inset and 6C upper right inset), and occasionally in blood vessel endothelium (Figure 6C).

### LepR in the Ut/Pl and Ut

#### LepR gene expression during pregnancy and after aglepristone induced abortion

**Figure 7:**
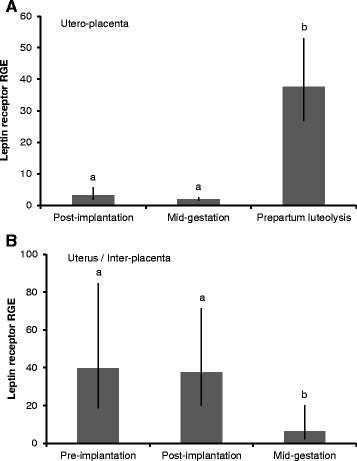
**Leptin receptor gene expression in the utero-placental and uterine compartments during pregnancy.** Leptin receptor gene expression during pregnancy in the utero-placental compartments (Ut/Pl) **(A)**, and in the pre-implantation uterus and inter-placental uterine sections (Ut) **(B)**. RGE: relative gene expression. Bars and whiskers present the geometric mean and the deviation factor. Bars with different superscripts differ at P < 0.001 (Ut/Pl) and at P ≤ 0.019 (Ut).

LepR expression in the Ut/Pl increased at the time of prepartum luteolysis compared to the post-implantation and mid-gestation stages (P < 0.001; Figure 7A). In the Ut, LepR mRNA concentrations were higher at pre- and post-implantation than at mid-gestation (P ≤ 0.019; Figure 7B). LepR expression was significantly down-regulated in the Ut/Pl compared to the Ut at post-implantation (P = 0.003) and at mid-gestation (P = 0.043; Figure 2B).

After aglepristone treatment, LepR mRNA levels in the Ut/Pl and Ut (Figure 3B) were similar at all time-points compared to the mid-gestation stage (P = 0.06 and P = 0.63, respectively).

### Immunohistochemical detection of LepR during pregnancy

Strong positive staining for LepR was visible in the uterine surface epithelium, and the superficial and deep uterine glands also showed positive reaction. Stromal signals were evident in the endometrium close to the lumen, especially in the pre-implantation group (Figure 4B). In later pregnancy stages, the glandular epithelium stained relatively more intensely than at pre-implantation (Figure 4D).

In the Ut/Pl, LepR signals were evident in fetal trophoblast cells of the placental labyrinth, while maternal decidual cells stained more weakly at all pregnancy stages (Figure 5B,D). LepR immunoreactivity was especially strong in the trophoblasts invading large maternal vessels at the base of the labyrinth (Figure 5D, inset). In the glandular chambers, LepR protein was detected in the epithelial cells as well as in the stroma (Figure 5F). Deep uterine glands of the Ut/Pl also showed positive signals (not shown).

The myometrium (Figures 4B upper right inset and 5F inset), blood vessel media, and sporadically also blood vessel endothelial cells (Figures 4D and 5B,D), all stained positive for LepR in the Ut and Ut/Pl.

### In situ hybridization for LepR

In the post-implantation Ut, LepR mRNA was present in the surface and glandular epithelial cells with strong signals in the endometrial stroma close to the luminal epithelium (Figure 6B).

In the Ut/Pl at prepartum luteolysis, strong LepR mRNA expression was detected in the fetal trophoblasts of the placental labyrinth (Figure 6D), while decidual cells (Figure 6D) and epithelial cells of the glandular chambers and deep uterine glands showed weaker reactivity. Positive signals were also present in the myometrium (Figure 6B upper right inset and Figure 6D upper right inset), and sporadically in blood vessel endothelial cells (Figure 6D).

## Discussion

We found that in the Ut, LepR expression was up-regulated at pre- and post-implantation compared to mid-gestation but Lep expression did not change. Additionally, LepR and Lep were co-localized in the glandular epithelium and in the luminal epithelial cells of the Ut, which is in direct contact with the early canine embryo. In the studies of Kitawaki et al. [57] and Cervero et al. [5], LepR expression in the human endometrium increased gradually from the proliferative to the early or mid-late secretory phase coinciding with the time of uterine receptivity and implantation. Protein signals for LepR and Lep in the uteri of women were predominantly localized in the luminal epithelium, in the endometrial glands and in stromal cells [3], which is in accordance with our study. Bartel et al. [50] also described immunoreactivity for both proteins in the surface and glandular epithelium, and occasional Lep staining in the stromal parts of the endometrium during diestrus and anestrus in non-pregnant dogs, but the date of ovulation was not known. In contrast to our study, Schäfer-Somi et al. [37] and Beceriklisoy et al. [39] did not detect Lep mRNA expression by qualitative PCR in the uterus of non-pregnant bitches in early diestrus or in pregnant dogs at pre-implantation and at d 20–35 of gestation. In the same study [37], Lep mRNA was not found in pre-implantation canine embryos either, while Lep protein and LepR protein and mRNA expression were not evaluated. The discrepancy between these reports and ours regarding uterine and utero-placental Lep expression may be due to methodology, because the semi-quantitative real-time (TaqMan) PCR used here is more sensitive than the qualitative approach. The expression/secretion of downstream molecular markers of endometrial receptivity, *e.g.*, adhesion molecules (β3 integrin) and cytokines (LIF, IL-1 and their receptors), was also increased after treatment of human endometrial epithelial cells with Lep [25,26]. In bitches, the expression of integrins is higher in the uterus of early pregnant than reproductive stage-matched non-pregnant diestrous animals [58]. Our findings therefore suggest that the Lep signaling pathway may be involved in the early embryo-maternal cross-talk in the dog.

A role for LepR in the canine implantation/placentation process may be hypothesized after our study found decreased LepR mRNA levels at placentation sites compared to the inter-placental uterus. In mice, LepR down-regulation at implantation sites was previously shown by global gene expression analysis [59], and Yoon et al. [60] also detected increased LepR expression at inter-implantation compared to implantation sites in mouse uteri. This hypothesis is further supported by the results of a recent study [61] in which women with recurrent implantation failure had lower endometrial Lep and higher LepR expression compared to fertile controls. These data indicate that, as in other species, decreased LepR expression at the placentation site may also be crucial for successful implantation and placentation in bitches.

Increased Lep expression in the Ut/Pl at post-implantation compared to mid-gestation may further point to a possible role for Lep during implantation and placenta formation in the dog. The localization of Lep and LepR protein and mRNA in fetal trophoblast cells in our study is in agreement with previous data on women, mice, pigs and cats [22,62-67], and may imply an autocrine/paracrine effect. The positive role of Lep was evidenced previously in human and murine models *in vitro*, where Lep promoted fetal trophoblast cell invasion *via* MMP-2 and −9, and Lep also showed its mitogenic potential [22-24,68,69]. Taken together, a regulatory, perhaps stimulatory role for Lep on trophoblast cell migration and proliferation during the process of canine implantation and placentation may be likely however this cannot be definitely concluded from our data.

Even though the reason for the prepartum up-regulation of utero-placental Lep and LepR is still not clear, it could imply a physiological role in canine parturition. Lep is a pro-inflammatory cytokine showing structural similarities to the type I cytokine superfamily [70] and may have an immune-modulatory function before and during labor. In women, serum Lep levels were positively correlated with other cytokines, *e.g.*, IL-6, interferon-ɤ-inducible protein and C-reactive protein in late pregnancy [71]. During induced parturition, plasma Lep concentrations increased and were higher in women after spontaneous delivery than after Cesarean section without labor. Similarly, Lep expression in the placenta after natural birth was also higher than after Cesarean section [72]. A stimulatory effect of Lep on pro-inflammatory mediators was shown by Lappas et al. [73], where prostaglandin F2α (PGF2α), prostaglandin E2, IL-1β, IL-6 and TNFα release was increased after *in vitro* treatment of term human placentas with Lep. In dogs, the source of the PGF2α rise before birth seems to be the placenta, where enzymes of prostaglandin biosynthesis (cyclooxygenase 2, prostaglandin E synthase) are up-regulated prepartum [41]. In our study, the significant increase in Lep and LepR expression in the Ut/Pl at prepartum luteolysis and their localization within those cells, where members of the prostaglandin synthesis pathway were also found [41], may suggest an autocrine/paracrine regulatory role for Lep in the events leading to parturition in the bitch.

The glandular epithelium was also a site of Lep and LepR expression both in the Ut and Ut/Pl. An autocrine/paracrine function regulating endometrial secretory capacity and thus histiotrophe production may be likely.

In pigs, myometrial expression of Lep and LepR transcript and protein were detected both in the luteal phase of the cycle and during pregnancy [65,66]. We also found Lep and LepR in the muscle layer of the canine uterus during all pregnancy stages, and therefore it seems that they are normal constituents of the myometrium. Because in our experiments full thickness Ut and Ut/Pl tissues were used, the degree of independent contribution of the myometrium, endometrium or placental labyrinth to the detected changes in Lep and LepR gene expression levels cannot be ascertained without further compartmentalization studies. Certain tissue layers may have contributed differently at given time points over the course of gestation, according to their specific role in the endocrine and physiological events, *e.g.,* during implantation or parturition. Furthermore, an increasing mass of the placental labyrinth with advancing pregnancy could have altered, at least to some extent, the ratio of the uterine compartments at placentation sites.

The LepR antibody used here identifies both the long and short isoforms of the receptor, which differ in their ability of signal transduction. While all transmembrane receptor isoforms can activate the tyrosine kinase Janus kinase (JAK2), only the long isoform with a full-length intracellular domain has signal transducer and activator of transcription (STAT)-binding sites and is able to activate the main signalling cascade JAK2/ STAT3. However, the short isoforms are also capable of signalling through various intracellular pathways and mediate Lep’s actions (reviewed by [74,75]). Therefore, the long and short LepR isoforms are relevant to investigate even though the long isoform is of particular interest, and studies on the expression/function of the different LepR splice variants in the dog could be planned.

In a few animals in our study, Lep and/or LepR mRNA expression was not detected in the Ut or Ut/Pl, which might have been due to degradation of RNA, sensitivity of our assay, individual differences in expression levels or pregnancy stage.

## Conclusions

The canine uterus and placenta are sources of Lep and targets of its actions during gestation. Lep and LepR are differentially expressed in these tissues over the course of pregnancy, and their presence in various cells types indicates multiple paracrine/autocrine functions. Lep and LepR are expressed both in the fetal and maternal sides of the placenta; thus, a role in placental physiology and feto-maternal cross-talk seems likely. The Lep signaling system may be one of the pathways involved in the establishment and maintenance of pregnancy, and may also play a regulatory role in parturition in the bitch.
